# Gut Microbiota Mediates the Preventive Effects of Dietary Capsaicin Against Depression-Like Behavior Induced by Lipopolysaccharide in Mice

**DOI:** 10.3389/fcimb.2021.627608

**Published:** 2021-04-27

**Authors:** Jing Xia, Li Gu, Yitong Guo, Hongyan Feng, Shuhan Chen, Jessore Jurat, Wenjing Fu, Dongfang Zhang

**Affiliations:** Department of Pharmacognosy, School of Pharmacy, China Medical University, Shenyang, China

**Keywords:** capsaicin, depression, gut microbiota, inflammation, lipopolysaccharide

## Abstract

Capsaicin (CAP) is an active ingredient in chili pepper that is frequently consumed. It exerts various pharmacological activities, and also has potential effects on mental illness. However, its mechanism of antidepressant effects is still unclear. Based on the emerging perspective of the gut-brain axis, we investigated the effects of dietary CAP on gut microbes in mice with depression-like behaviors induced by lipopolysaccharide (LPS). C57BL/6J male mice (four weeks old) were given specific feed (standard laboratory chow or laboratory chow plus 0.005% CAP) for 4 months. During the last five days, LPS (0.052/0.104/0.208/0.415/0.83 mg/kg, 5-day) was injected intraperitoneally to induce depression. Behavioral indicators and serum parameters were measured, and gut microbiota were identified by sequencing analysis of the 16S gene. This study showed that dietary CAP improved depressive-like behavior (sucrose preference test, forced swimming test, tail suspension test) and levels of 5-HT and TNF-α in serum of LPS-induced mice with depression-like behaviors. In addition, CAP could recover abnormal changes in depression-related microbiota. Especially at the genus level, CAP enhanced the variations in relative abundance of certain pivotal microorganisms like *Ruminococcus*, *Prevotella*, *Allobaculum*, *Sutterella*, and *Oscillospira*. Correlation analysis revealed changes in microbiota composition that was closely related to depressive behavior, 5-HT and TNF-α levels. These results suggested that dietary CAP can regulate the structure and number of gut microbiota and play a major role in the prevention of depression.

## Introduction

Depression is an emotional dysfunction caused by abnormalities in the individual’s genetic system or by changes in the environment (Hecke et al., 2017; Bleys et al., 2018). It manifests as depressed mood, slow thinking, impaired cognitive function, negative and pessimistic thoughts, and even suicidal tendencies. Morbidity of major depressive disorder (MDD) is increasing year by year (Winter et al., 2018), and it is the second most common disease threatening human health at present. Simultaneously, it is more expensive to treat and causes more damage to the human body compared with other mental diseases (Wang et al., 2010; Otte et al., 2016). Numerous studies have demonstrated that the gastrointestinal microbiota can influence mood, including depression and anxiety (Bravo et al., 2010; Opie et al., 2015), and even brain development (Heijtz et al., 2011) *via* neuroendocrine, neuroimmune, neural and humoral pathways (Cryan and Dinan, 2012; Dinan and Cryan, 2013). There are clinical studies showing that the structure of gut microbiota in MDD patients has altered observably, and that bacterial abundance is affected by depression, showing some degree of decline (Chen et al., 2018). In addition, the stability of gut microbiota is greatly affected by diet style, and diet can alter the amount and structure of gut microbes, which in turn affects mood and brain activity (Paola et al., 2010; Giada et al., 2017).

Chili pepper (*Capsicum annuum* L.) is an important vegetable or spice and widely consumed around the world (López-Carnllo et al., 1994). The main active components in chili pepper are alkaloids, such as capsaicin, nordihydrocapsaicin and dihydrocapsaicin (Barbero et al., 2008). Capsaicin (CAP), the main active ingredient of chili pepper, has multiple pharmaco-activities and therapeutic applications. These include its function as an anti-inflammatory and analgesic, an antioxidant, a controller of blood pressure, a regulator of blood lipids, and other pharmacological effects (Jolayemi and Ojewole, 2013; Vianna et al., 2018; Sánchez et al., 2019; Segawa et al., 2019). Additionally, it activates TRPV1 and initiates a complex cascade of reactions, including neuronal activation, release of proinflammatory mediators and receptor desensitization. Many immunocytochemical studies have shown that TRPV1 is expressed in multiple brain regions (Mascarenhas et al., 2015). All studies show that they may play a crucial part in controlling anxiety and other emotional responses in addition to their function as pain detectors (Terzian et al., 2009). Studies have also found that repeated oral administration of CAP demonstrates anti-anxiety effects in specific rat models and it also can protect hippocampal synaptic plasticity and spatial memory retrieval *via* the TRPV1 channel (Li et al., 2008; Choi et al., 2015). Previous research has found that CAP significantly increased the *Firmicutes*/*Bacteroidetes* ratio in obese diabetic mice at the phylum level and also regulated other levels of gut microbiota (Song et al., 2017). Another article showed that dietary CAP led to higher levels of microorganisms associated with butyrate production and lower levels of lipopolysaccharide (LPS)-producing microbiota (Kang et al., 2017). These suggest that CAP can function to improve the health of the gut microbiome.

However, there has been little research on whether capsaicin can prevent the effects of depression. Lipopolysaccharide can cause depressive behavior in animals by causing neuroinflammation, and this model is also widely used in depression research. At the same time, the importance of gut microbiota in LPS-induced depression models has gradually attracted researchers’ attention (Huang et al., 2019; Zhang et al., 2020). Based on previous reports, the purpose of our study was to verify that dietary CAP can effectively prevent behavioral disorders such as anxiety, depression, disgust and despair caused by LPS. The effects of CAP on neuroinflammation and the levels of the monoamine neurotransmitter 5-hydroxytryptamine (5-HT) were investigated on the side. The diversity and abundance of the gut microbiome in mice was further analyzed by 16S rRNA sequencing to elucidate that CAP can improve microbiome composition and regulate depressive behavior.

## Materials and Methods

### Experimental Animals

Four-week-old male C57BL/6J mice (bodyweight: 16-18g) were purchased from Liaoning Changsheng biotechnology co., Ltd (Shenyang, China). The animal study was carried out in accordance with the Guideline for Animal Experimentation of China Medical University.

The mice were single housed to prevent cross contamination of gut microbiota in specific pathogen-free animal facilities (maintained at 20-25°C, 50-55% relative humidity, and a 12/12-h light/dark cycle). They were provided with access to a laboratory diet and water ad libitum. Mice were acclimatized for one week to the experimental environment before the actual experiment.

### Experimental Groups and Diet

The mice were randomly allocated into four groups (n=6 per group): (1) Control group (CON); (2) Lipopolysaccharide-treated group (LPS); (3) Capsaicin diet group (CAP); (4) Capsaicin diet + lipopolysaccharide-treated group (CAP+LPS). The mice of Control and LPS groups were fed standard laboratory chow (Department of Laboratory Animal Science of China Medical University). The mice in the CAP and CAP+LPS groups were fed standard laboratory chow plus 0.005% capsaicin (Targetmol, Boston, MA, USA). All mice were fed for 4 months. During the last 5 days, mice in LPS and CAP+LPS groups received lipopolysaccharide (Beijing Solarbio Science & Technology Co., Ltd., Beijing, China) once daily *via* intraperitoneal injection. The following doses of LPS were used according to previous methods: 0.052/0.104/0.208/0.415/0.83 mg/kg (5-day) (Wickens et al., 2017). The CON and CAP groups were injected with an equal volume of saline in the same manner. Body weight was measured after behavioral testing.

### Behavior Tests

#### Open Field Test

Independent locomotor activity and anxiety-like behaviors were assessed by the open field test (OFT) (Willner et al., 1992). The instrument consisted of an open box (50 × 50 × 55 cm) with a white surface over the bottom. The bottom is divided into 9 equal squares. After 6 min of acclimatization, the mice were tested for 6 min to record the number of crossings and rearings.

#### Sucrose Preference Test

The Sucrose Preference Test (SPT) is an important method to detect anhedonia. It was performed by reference to the previous literature (Mao et al., 2009; Ma et al., 2011). Before the test, the mice underwent a 72-hour adaptation period. For this period, mice were trained to adapt to sucrose solution with two bottles of 1% (w/v) sucrose solution placed in each cage. After 24 hours, sucrose solution in one bottle was replaced with pure water for 24 hours. On the night of the last LPS injection, all mice were deprived of water and food for 12h. On the following morning, they were individually placed in cages with free access to two bottles containing water and 1% sucrose, and intake of water and 1% sucrose was measured over two hours. The sucrose preference index was calculated as the percentage of 1% sucrose solution intake relative to total liquid intake.

#### Forced Swimming Test

The forced swimming test (FST) was used to measure the depressive behavioral changes. The mice were subjected to the FST, which was similar to that described in previous studies with minor modification (Porsolt et al., 1978). The mice were placed in a transparent glass cylinder (21 cm in height, 16.5 cm in diameter) filled with 13 cm-deep water at 24 ± 1°C for 6 min. The mice were forced to swim for 6 minutes and their time spent immobile was observed for the last 4 minutes. The mice were defined to be immobile when their head was above the water without significant limb motion. An experimenter blinded to the purpose of the experiment recorded the results of immobility time.

#### Tail Suspension Test

The tail suspension test (TST) was used to assess behavioral despair. TST was implemented according to a previous method (Ma et al., 2011). Briefly, mice were suspended by their tails with tape in a position about 1cm away from the tail tips and its head 60 cm above the floor. The test lasted for 6 minutes and the total duration of immobility time in the final 4 min was recorded. Immobility refers to the time the mouse hung passively or remained completely motionless. Immobility time was noted by an observer blinded to the purpose of the experiment.

### Sample Collection and Preparation

After three behavior tests, feces samples from each mouse were collected, transferred to EP tubes, and stored at -80°C for later analysis. Blood samples were obtained after enucleating the eyeballs and centrifuged at 5000 rpm and 4°C for 10 min. Serum samples were collected and stored at -80°C for ELISA analysis.

### ELISA Measurement

The 5-HT and TNF-α levels in the serum were determined by specific ELISA kit (Cusabio, Houston, TX, USA; https://www.cusabio.com/ and Thermo Fisher Scientific, Waltham, MA, USA) according to the manufacturers’ instructions.

### 16S rRNA Sequencing Analysis

Total bacterial genomic DNA samples were extracted using the OMEGA Soil DNA Kit (D5625-01), according to the manufacturer’s instructions, and stored at -20°C prior to further analysis. The quality and quantity of extracted DNA were detected using a NanoDrop ND-1000 spectrophotometer (Thermo Fisher Scientific, Waltham, MA, USA) and agarose gel electrophoresis, respectively. PCR with Q5 High-Fidelity DNA polymerase and primers 338F (5’-ACTCCTACGGGAGGCAGCA-3’) and 806R (5’-GGACTACHVGGGTWTCTAAT-3’) was used to amplify the V3-V4 region of the 16S rRNA gene. The resulting PCR amplicons were purified with Agencourt AMPure Beads (Beckman Coulter, Indianapolis, IN) and quantified using the PicoGreen dsDNA Assay Kit (Invitrogen, Carlsbad, CA, USA). After the individual quantification step, amplicons were pooled in equal amounts, and pair-end 2×300 bp sequencing was performed using the Illlumina MiSeq platform with MiSeq Reagent Kit V3 at Shanghai Personal Biotechnology Co., Ltd (Shanghai, China).

The Quantitative Insights Into Microbial Ecology (QIIME, v1.8.0) pipeline was employed to process the sequencing data, as previously described. Briefly, raw sequencing reads with exact matches to the barcodes were assigned to respective samples and identified as valid sequences. The low-quality sequences were filtered through following criteria: sequences that had a length of <150 bp, sequences that had average Phred scores of <20, sequences that contained ambiguous bases, and sequences that contained mononucleotide repeats of >8 bp. Paired-end reads were assembled using FLASH. After chimera detection, the remaining high-quality sequences were clustered into operational taxonomic units (OTUs) at 97% sequence identity by UCLUST (Edgar, 2010). A representative sequence was selected from each OTU using default parameters. OTU taxonomic classification was conducted by BLAST searching the representative sequences set against the Greengenes Database using the best hit.

α-diversity was visualized according to the rarefaction curve and principal coordinate analysis (PCoA), and orthogonal partial least squares discriminant analysis (OPLS-DA) was performed to analyze β-diversity. The linear discriminant analysis (LDA) effect size (LEfSe) was further used to identify the dominant phylotypes responsible for differences between CON, LPS and CAP+LPS groups.

### Statistical Analysis

Data are reported as mean ± SEM of at least three independent experiments. To assess potential correlations between parameters, Spearman’s correlation analysis was applied using RStudio software (Boston, MA, US). SPSS version 19.0 was used for all statistical analysis. The data were statistically analyzed using one-way analysis of variance (ANOVA). If p < 0.05, the difference was considered statistically significant.

## Results

### Effects of Dietary CAP on Body Weight and Depression-Like Behavior in LPS-Treated Mice

**Figure 1:**
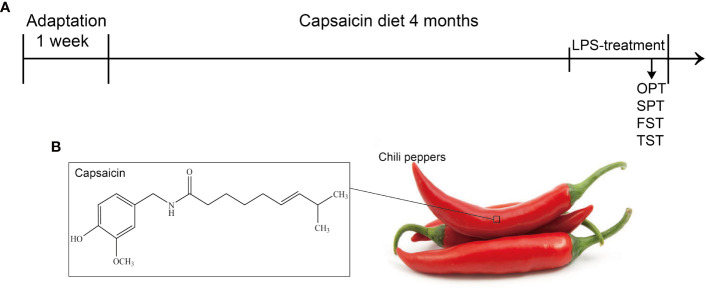
**(A)** Schematic procedure of the experiments: SPT, sucrose preference test; FST, forced swimming test; TST, tail suspension test; **(B)** The chemical structure of CAP isolated from chili peppers.

**Figure 2:**
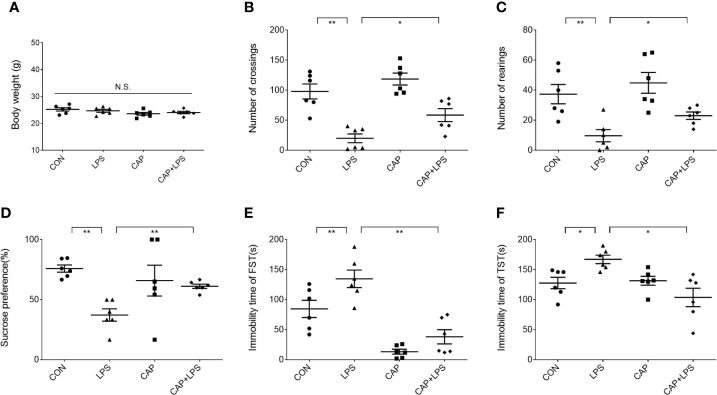
Effects of dietary CAP on body weight and depression-like behavior in LPS-treated mice. **(A)** Body weight (F_3,20_ = 1.771, P > 0.05). **(B)** Number of crossings in open field test (F_3,20_ = 17.99, P < 0.01). **(C)** Number of rearings in open field test (F_3,20_ = 8.728, P < 0.01). **(D)** Sucrose preference of sucrose preference test (F_3,20_ = 5.296, P < 0.01). **(E)** Immobility time of forced swimming test (F_3,20_ = 20.05, P < 0.01). **(F)** Immobility time of tail suspension test (F_3,20_ = 6.411, P < 0.01). Results are represented as mean ± SEM (n = 6). Asterisk has been used for significant differences (*P < 0.05, **P < 0.01, N.S., not significant).

Mice in each group were given a specific chow for four months (**Figure 1A**), and the CAP and CAP+LPS groups mice in particular were given a diet containing CAP (**Figure 1B**). During the last five days of the specific diet, mice were injected with LPS to induce depression-like behavior. To explore the effects of CAP on LPS-induced depressed behavior, body weight was measured before and after the onset of depressive behavior and the open field test, sucrose preference test, forced swimming test, and tail suspension test were adopted to evaluate. Results show that there was no significant change of body weight in each group (**Figure 2A**). In the open field test, the number of crossings and rearings in the LPS group were lower than those in the CON group (P < 0.01) (**Figure 2B**), and these values were effectively improved in the CAP+LPS group (P < 0.05) (**Figure 2C**). The sucrose preference index of the LPS group decreased significantly (P < 0.01), but this decrease was rescued in the CAP+LPS group (P < 0.01) (**Figure 2D**). Immobility time in the FST of LPS group mice increased more than in the CON group (P < 0.01). CAP+LPS treatment effectively reduced this indicator (P < 0.01) (**Figure 2E**). In the tail suspension test, the immobility time of LPS-treated mice was significantly prolonged (P < 0.05), and CAP diet can decrease it significantly (P < 0.05) (**Figure 2F**).

### Dietary CAP Prevented Alterations in Serum 5-HT and TNF-α Levels in LPS-Treated Mice

**Figure 3:**
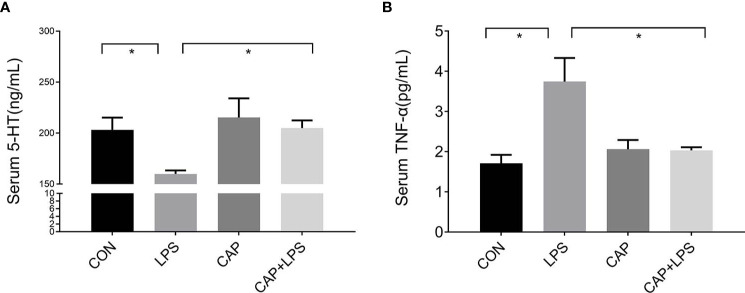
Dietary CAP prevented alterations in serum 5-HT and TNF-α levels in LPS-treated mice. **(A)** 5-HT (F_3,16_ = 4.259, P < 0.05). **(B)** TNF-α (F_3,20_ = 7.436,P < 0.01). Data are represented as mean ± SEM (n = 5). Asterisk has been used for significant differences (*P < 0.05).

The 5-HT and TNF-α levels of serum from each group were detected, respectively. 5-HT levels of the LPS group were prominently reduced (P < 0.05) compared to the CON group. This reduction in serum 5-HT levels was attenuated in the CAP+LPS group (P < 0.05) (**Figure 3A**). TNF-α levels in LPS-treated mice were significantly increased (P < 0.05), and this increase was also attenuated in the CAP+LPS group (P < 0.05) (**Figure 3B**).

### Dietary CAP Improved the Gut Microbiota Structure in LPS-Treated Mice

**Figure 4:**
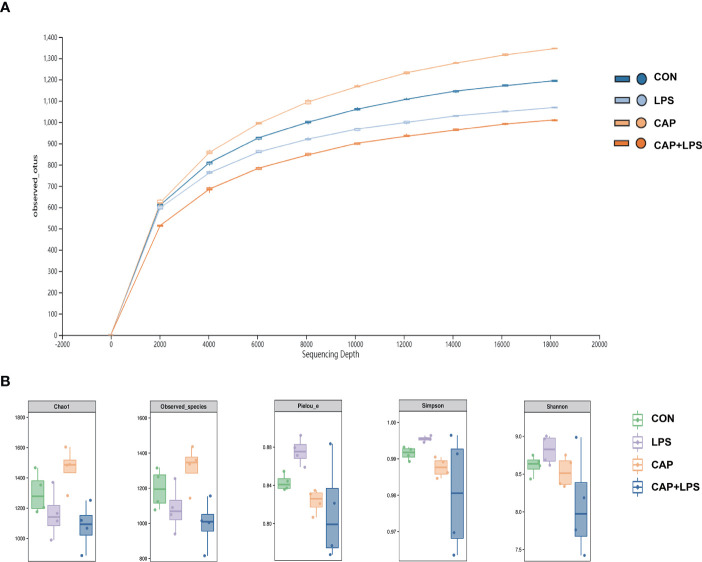
**(A)** The rarefaction curve to assess the sequencing depth in every group (n = 4); **(B)** Chao1 estimator, Observed species, Pielou’s index, Simpson index, Shannon index (n = 4). Data are presented as box and whisker plots. The box denotes the interquartile range (IQR, 75th to 25th percentiles of the data), and the mean value is represented by a straight line within the box; whiskers extend to 1.5 × IQR, or the most extreme value. The Kruskal-Wallis test was used to conduct one-way ANOVA on the results. And there were no significant differences among CON, LPS and CAP+LPS groups.

**Figure 5:**
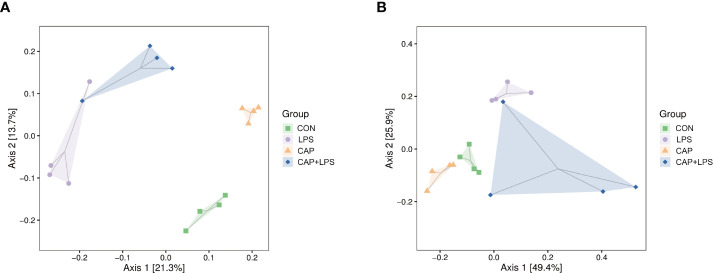
**(A)** Unweighted UniFrac distance-based principal coordinate analysis (PCoA) (n = 4); **(B)** Weighted PCoA (n = 4).

**Figure 6:**
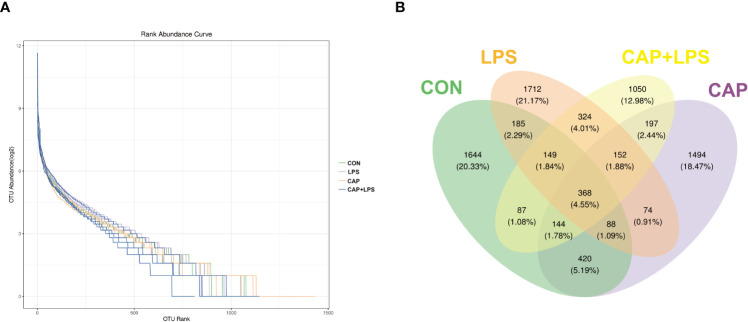
**(A)** Rank abundance curves in each sample (n = 4); **(B)** Venn diagram depicting richness and overlap of OTU in four groups (n = 4).

To investigate whether dietary CAP and LPS treatment resulted in specific changes of gut microbiota structure, the gut microbiota of mice at 4 months after specific feeding was evaluated by 16S rRNA sequencing analysis. Results of rarefaction curves for each group show that the mean value for number of OTUs observed at various sequencing depths. (**Figure 4A**). α-diversity analysis shows the richness, diversity and evenness of the microbiome composition among four groups. In the LPS group compared to the CON group, there were no significant differences in the richness (using the Chao1 and Observed species indices as representatives), diversity (using the Shannon and Simpson indices as representatives) and evenness (represented by Pielou’s index), and there were no obvious differences between LPS and CAP+LPS groups as well (**Figure 4B**). To further understand similarities in microbial composition between individuals, multidimensional cluster analysis was performed. Both unweighted and weighted UniFrac PCoA analysis indicated an obvious clustering among different groups (**Figures 5A, B**). In the OTU rank curve chart, the curve of the CAP+LPS group was steeper than that of other groups. This indicates that the microbiome of the CAP+LPS group had a relatively low evenness (**Figure 6A**). According to the Venn diagram, we can see that 790 and 993 OTUs were shared between the CON and LPS groups and between the LPS and CAP+LPS groups, respectively (**Figure 6B**).

### Dietary CAP Improved the Gut Microbiota Composition in LPS-Treated Mice

**Figure 7:**
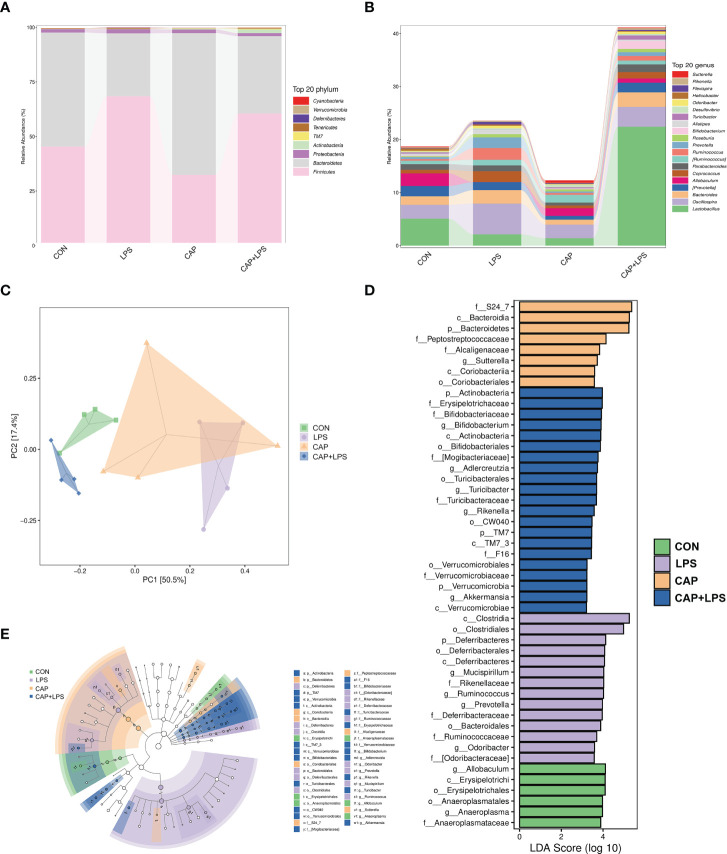
**(A)** The community structures of different microbes at the phylum level in the four groups, colors indicate distinct microbe of phylum (n = 4); **(B)** The microbiota structures at the genus level in the four groups, different colors denote distinct microbe of genus (n = 4); **(C)** Orthogonal partial least squares discriminant analysis (OPLS-DA) of the microbiota in all groups (n = 4); **(D)** Linear discriminant analysis effect size (LEfSe) 10 was set up with 0.05 as the alpha value for Kruskal-Wallis and Wilcoxon test and then selected OTUs with LDA scores above 3 (n = 4); **(E)** LDA scores greater than 3 when comparing all groups, as described above (n = 4).

**Figure 8:**
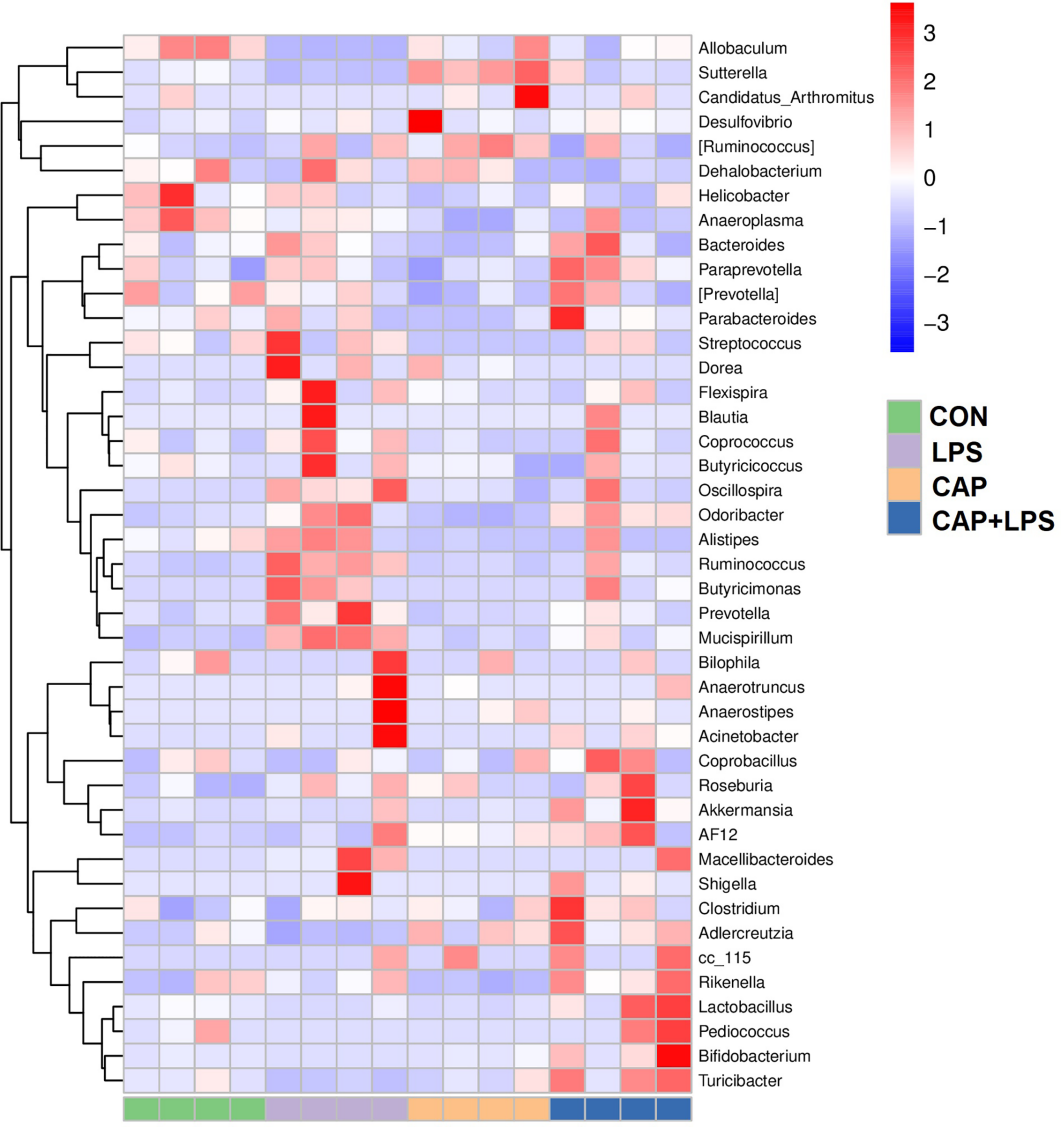
The heat map of microbe relative abundance of the genus (n = 4). Colors indicate the values (high values in red, low in blue).

To determine the specific constitution of gut microbiota in each group, we analyzed the abundance of microbiota at genus and phylum levels. From relative abundance of microbiota at the phylum level, we can see that abundance of *Firmicutes*, *Proteobacteria* and *Deferribacteres* in the LPS group were increased, but abundance of *Bacteroidetes* and *Actinobacteria* were decreased. However, CAP+LPS treatment can effectively regulate this abnormal change (**Figure 7A**). At the genus level, abundance of *Allobaculum*, *Turicibacter*, *Adlercreutzia* in the LPS group were reduced compared with CON mice. Meanwhile, the relative abundance of *Ruminococus*, *Sutterella*, and *Adlercreutzia* were increased. Nevertheless, CAP+LPS treatment can effectively reverse these changes (**Figure 7B**). In addition, to understand the difference in microbiota abundance and composition between groups, orthogonal partial least squares discriminant analysis (OPLS-DA) was carried out. According to the results, we found that there is an evident microbiota composition clumped in the LPS group, distinguished from the CON and CAP+LPS groups (**Figure 7C**). To probe the distinctions in gut microbes in the groups, LEfSe analysis was carried out to confirm the dominant communities causing the distinctions in the groups (**Figures 7D, E**). The highest distinctions (LDA score > 3.0) of microbiota (from phylum to genus) in all groups were distinguished. In the LPS group, the relative abundances of *Rikenellaceae* (p < 0.05), *Ruminococcaceae* (p < 0.05), *Clostridia* (p < 0.05), *Deferribacterales* (p < 0.01), *Ruminococcus* (p < 0.05), *Deferribacteres* (p < 0.01), *Deferribacteraceae* (p < 0.01), *Clostridiales* (p < 0.05), *Prevotella* (p < 0.05), *Mucispirillum* (p < 0.01), *Deferribacteres* (p < 0.01), *Odoribacter* (p < 0.01), *Odoribacteraceae* (p < 0.01) and *Bacteroidales* (p < 0.05) were obviously higher than those in CON and CAP+LPS group. However, in the CAP+LPS group, *Mogibacteriaceae* (p < 0.05), *Actinobacteria* (p < 0.01), *Bifidobacterium* (p < 0.05), *Akkermansia* (p < 0.05), *TM7_3* (p < 0.05), *Erysipelotrichaceae* (p < 0.05), *Turicibacter* (p < 0.05), *Verrucomicrobia* (p < 0.05), *Verrucomicrobiaceae* (p < 0.05), *Turicibacteraceae* (p < 0.05), *TM7* (p < 0.05), *CW040* (p < 0.05), *Bifidobacteriales* (p < 0.05), *Verrucomicrobiae* (p < 0.05), *Actinobacteria* (p < 0.05), *Bifidobacteriaceae* (p < 0.05), *Rikenella* (p < 0.05), *F16* (p < 0.05), *Adlercreutzia* (p < 0.05), *Turicibacterales* (p < 0.05) and *Verrucomicrobiales* (p < 0.05) were the most differentially abundant microbes. For in-depth study, we determined changes in gut microbiota in this model, clustered the microbes of the 50 genera with the highest relative abundance, and placed them on a heat map (**Figure 8**). Obviously, distributions in gut microbiota of these groups at the genus level are inconsistent. Compared with CON group, abundances of *Allobaculum*, *Sutteralla*, *Turicibacter*, *Adlercreutzia*, and *Bifidobacterium* were reduced in the LPS group, contrasted with a growth in abundance of *Oscillospira*, *Odoribacter*, *Mucispirillum*, *Prevotella*, and *Ruminococcus*, and dietary CAP could reverse the changes in these microbes. Among them, *Odoribacter* and *Mucispirillum* could be restored to the CON group level.

### Correlation Analysis Among Behavioral Indicators and Serum 5-HT, TNF-α Levels and Relative Abundances of Representative Gut Microbiota

**Figure 9:**
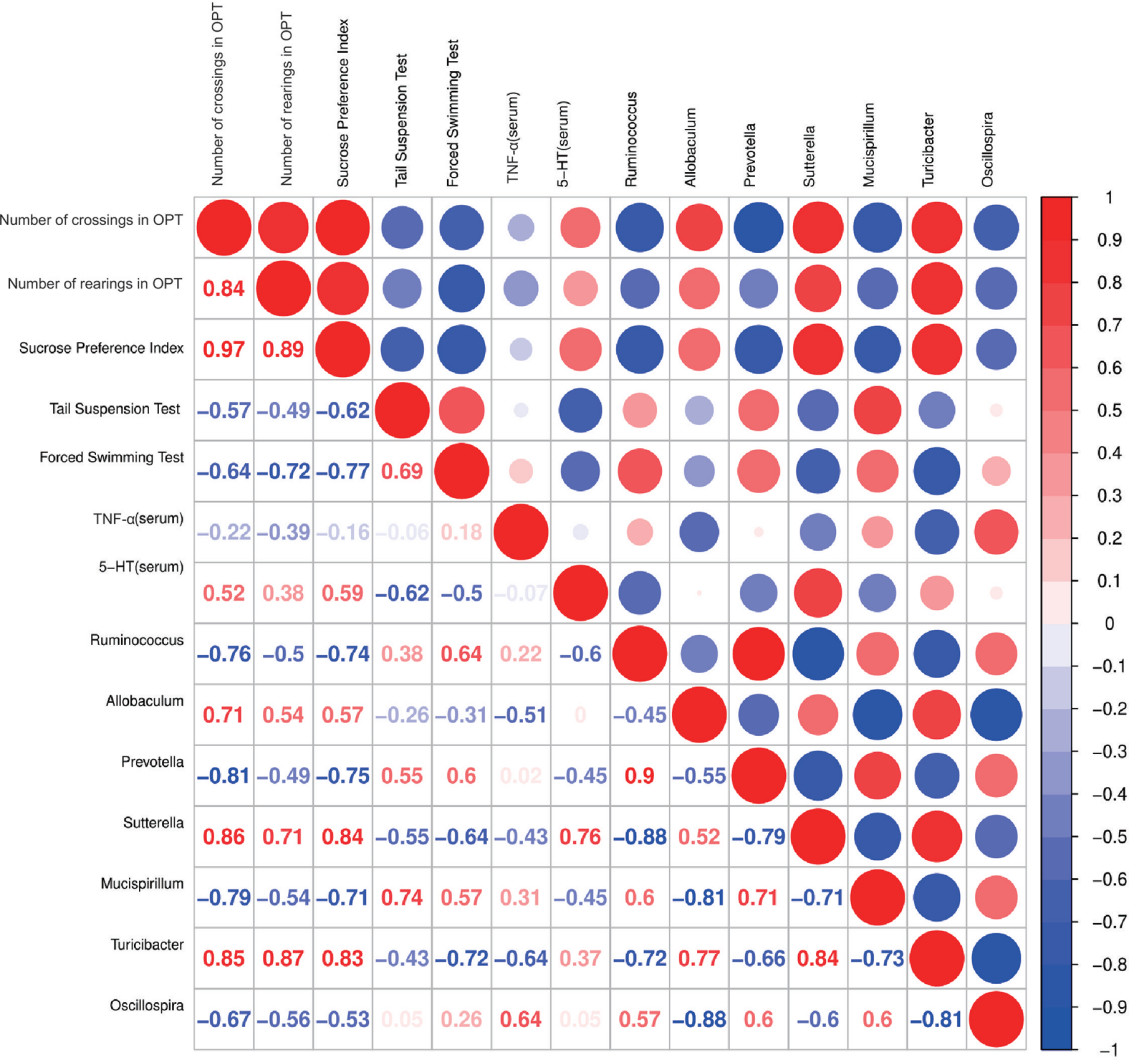
Correlation analysis between behavioral results, serum 5-HT, TNF-α levels and abundance of signature gut microbiota. Spearman’s correlation analysis was performed by data of the LPS group and CAP+LPS group (n = 8). In the upper right corner, the color and size of the circle denote the strength of correlation, red is positive, and blue is negative. The corresponding correlation numeric data could be found in the lower left corner.

Spearman’s correlation analysis indicated that number of crossings in OPT was positively correlated with the abundance of *Sutterella* (r = 0.86, p < 0.05) and number of rearings in OPT was positively correlated with the abundance of *Turicibacter* (r = 0.87, p < 0.01). The sucrose preference index was correlated with the relative abundances of *Sutterella* (r = 0.84, p < 0.01) and *Turicibacter* (r = 0.83, p < 0.05) positively and *Ruminococcus* (r = -0.74, p < 0.05) and *Prevotella* (r = -0.75, p < 0.05) negatively. Immobility time in the tail suspension test was positively correlated with the abundance of *Mucispirillum* (r = 0.74, p < 0.05). However, immobility time in the forced swimming test was negatively correlated with the abundance of *Turicibacter* (r = -0.72, p < 0.05). Furthermore, the 5-HT levels in serum were positively correlated with the abundance of *Sutterella* (r = 0.76, p < 0.01) (**Figure 9**).

## Discussion

In our study, we found that dietary CAP significantly improved depression-like behaviors caused by LPS, such as anhedonia and hopelessness. In addition, LPS can disrupt the composition and structure of gut microbiota, resulting in decreased levels of 5-HT, a neurotransmitter, and excessive levels of TNF-α, an inflammatory factor, in serum. However, CAP can regulate the serum levels of 5-HT and TNF-α in LPS-treated mice. Of note, CAP can effectively improve the abundance and type of gut microbiota, and is additionally highly related to behavioral change.

Behavioral differences are the most intuitive symptom manifestations of depression models (Ma et al., 2011). In the SPT, FST and TST, LPS-treated mice showed signs of anhedonia and hopelessness, which the mice given dietary CAP effectively avoided. Inflammatory cytokines like IL-6 and TNF-α are involved in the pathogenesis of depression and have been found to increase in previous animal models of depression and in patients with clinical depression (Karson et al., 2013; Yu et al., 2018). TNF-α is a pro-inflammatory factor, which is the earliest cytokine produced in a series of inflammatory reactions. It can promote the secretion of neurotrophic factor, and contribute to enhancing the adhesion of neurons and axon growth (Annibalini et al., 2014; Shen et al., 2015; Liu et al., 2016). Elevated levels of TNF-α caused by LPS weaken its effect on neurons, but CAP can prevent this. The mechanism of depression remains unclear, but the monoamine hypothesis is widely accepted (Liu et al., 2020). Most current treatments for depression also aim to enhance levels of monoamine neurotransmitters. Antidepressants such as fluoxetine hydrochloride work by boosting serotonin levels in the brain (Jazayeri et al., 2008). 5-HT is a representative monoamine neurotransmitter implicated in regulating several physiological activities and behaviors, including those related to emotion and anxiety, and low levels of 5-HT have turned out to be associated with depression (Pascucci et al., 2007; Taylor and Munafo, 2016; Wang et al., 2016). In this study, LPS caused mice to have lower 5-HT levels, but mice given dietary CAP did not experience the same decline.

In recent years, the gut microbiome has received great attention, and studies have revealed that gut microbiota are related to multiple diseases like depression, obesity, diabetes, and cancer (He et al., 2015; Bokan and Hauser, 2018; Cândido et al., 2018; Meng et al., 2018; Taylor and Holscher, 2018; Carlessi et al., 2019; Caspani et al., 2019; Prosperi, 2020). In the meantime, most studies showed that differences in diet can affect health and lead to corresponding changes in gut microbiota (Baffoni et al., 2011; Gaggia et al., 2011; Gioia et al., 2014; Nagpal et al., 2014; Walter, 2015; Nagpal et al., 2016; Sonnenburg et al., 2016; Dubinkina et al., 2017). CAP is a common alkaloid in human diets and is extensively consumed (Tyakht et al., 2012). Kang et al. found that different doses of CAP in the diet altered the gut microbiota of healthy subjects for two weeks. The results showed that CAP boosted the *Firmicutes*/*Bacteroidetes* ratio and the number of *Faecalibacterium* (Zhao et al., 2020). This suggests that CAP can regulate the composition of the microbiome, and several studies have shown that it can improve related diseases *via* regulation of microbes. For example, CAP enhanced levels of *Ruminococcaceae* and *Lachnospiraceae* associated with butyrate production, while it induced a decrease in the level of the key LPS-producing microorganism, S24-7, thus preventing chronic low-level inflammation (Kang et al., 2017). CAP also inhibits the growth of *Helicobacter pylori* thereby exerting a protective effect on gastroduodenal disease (Kang et al., 2016). In addition, CAP has therapeutic effects on diabetes *via* regulation of glucose homeostasis. This is related to its function of changing abundance of *Roseburia*, *Bacteroides* and *Parabacteroides* (Song et al., 2017). Previous studies have shown that CAP can improve symptoms such as anxiety and depression (Li et al., 2008; Choi et al., 2015; Song et al., 2017), but few studies have elucidated the mechanism of CAP to improve depression through the gut-brain axis from the microbial point of view.

Here, we probed mechanisms of fecal microbiota associated with dietary CAP in the prevention of depression with 16S rRNA gene sequencing. The result of PCoA analysis indicated obvious distinctions among the four study groups. To further investigate the effects of dietary CAP on depression-associated microbiota, we isolated the most prominent microbial changes in each group using LEfSe analysis and visualized the results using a heat map. At the phylum level, the difference in microbiota was chiefly reflected in the abundance of *Actinobacteria*, *Bacteroidetes*, *Deferribacteres* and *Firmicutes*. This work suggests that CAP-containing diets can distinctly improve LPS-induced differences in the type and abundance of gut microbiota. A decline in 5-HT levels, which is associated with several microorganisms, is a clear sign of depression. *Ruminococcus* belongs to phylum *Firmicutes*, order *Clostridiales* and the relative abundance of *Ruminococcus* is highly related to the production mechanism of 5-HT. *Ruminococcus* could produce tryptophan and prompt colonic enterochromaffin cells to produce and release 5-HT (Jones et al., 1997; Mawe and Hoffman, 2013). Most 5-HT in the body is produced from this colonic location. Subsequently, 5-HT enters the peripheral blood and plays a role in regulating emotions (Takaki et al., 1985; Yano et al., 2015). However, the abundance of *Ruminococcus* was abnormally increased in mice with depression-like behaviors, and serum 5-HT concentration, which was inversely related to *Ruminococcus* at this time, was thus significantly decreased (Allison et al., 2018; Lukić et al., 2019). In our results, *Ruminococcus* in the LPS group mice increased significantly, while the serum 5-HT levels decreased significantly, and dietary CAP could effectively regulate 5-HT and the abundance of *Ruminococcus* to restore both to their normal levels. Increased *Ruminococcus* may cause degradation of the mucus layer allowing other bacteria to infiltrate, leading to inflammation (Sean et al., 2015; Li et al., 2018). In addition, the changes in relative abundance of *Prevotella* can damage colonic epithelia, leading to inflammation and production of more inflammatory cytokines like IL-6, TNF-α and IL-8 (Berry et al., 2012). LPS caused variations in the abundance of microbiota related to inflammatory factors, and dietary CAP could effectively reverse changes in the abundance of the corresponding microbiota.

*Ruminococcus*, a phylum of gram-positive cocci, have been found to have a positive correlation to the DASS depression score in a previous study (Wang et al., 2017). At the same time, *Ruminococcus* in depressed men are significantly increased, which is related to their involvement in the process of tryptophan metabolism and the final regulation of tryptophan metabolites levels (Chahwan et al., 2019). Moreover, MDD patients also have changes in the amount of *Ruminococcus* in their guts (Lara et al., 2017; Cheung et al., 2019). After mice were treated with LPS, the quantitative changes of *Ruminococcus* were in accordance with previous studies, and dietary CAP could reduce this abundance. We hypothesized that CAP may affect tryptophan metabolism through *Ruminococcus*, thereby affecting the metabolites of tryptophan, such as 5-HT, and ultimately improving depressive behaviors. Additionally, *Prevotella*, a gram-negative anaerobic bacterium, are strongly associated with depressive symptoms (Xiao et al., 2014). A high abundance of *Prevotella* is often found in patients with MDD (Lin et al., 2017; Su et al., 2018), and abundance of *Prevotella* is also increased in depression-susceptible animals (Liu et al., 2016). Our results are consistent with these conclusions that LPS leads to a higher *Prevotella* level and CAP can mitigate this phenomenon.

Gut microbiota and 5-HT production go hand in hand. Few papers have investigated the relation between the antidepressant effects of CAP and its effect on gut microbial composition. Our correlation analysis showed a high correlation between serum 5-HT concentration and gut microbial abundance. Studies have indicated that brain damage could reduce the number of *Peptococcaceae*, but the proinflammatory cytokine CCL5 improved these numbers (Houlden et al., 2016; McGaughey et al., 2019). In inflammation-related diseases, the amount of *Oscillospira* was decreased (Elinav et al., 2011). In this study, we discovered that 5-HT levels have high and positive correlation with the relative abundance of *Sutterella* (r = 0.76, p < 0.01) in mice with depression-like behaviors. Thus, we inferred that gut microbes might be involved in 5-HT production through regulating the inflammatory response. Nevertheless, correlation analysis has limitations and could not reflect the causal relationship between the effects of CAP on depressed behavior and intestinal microbiota on neurotransmitter bioactivity. This requires further exploration in the future to probe underlying effects of CAP through the gut-brain axis.

Taken together, our data suggest that CAP may play a protective role in mice with depression-like behaviors, possibly through the regulation of gut microbiota.

## Conclusions

In conclusion, dietary CAP regulated the structure and the relative abundances of some key microbial species, such as *Ruminococcus* and *Prevotella*, to improve intestinal health, which in turn increased the levels of the monoamine neurotransmitter 5-HT, and reduced the levels of inflammatory cytokine TNF-α in LPS-induced mice with depression-like behaviors. Ultimately, it relieved depressive behaviors such as aversion to stimuli, anhedonia and hopelessness *via* the gut-brain axis. Although the underlying mechanisms of CAP on microbial metabolites and the gut-brain axis are still unclear, this research provides an explanation for the function of dietary CAP in preventing depression.

## Data Availability Statement

The datasets presented in this study can be found in online repositories. The names of the repository/repositories and accession number(s) can be found below: NCBI- SRA, accession number: PRJNA678526.

## Ethics Statement

The animal study was reviewed and approved by the Laboratory Animal Welfare and Ethics Committee of China Medical University.

## Author Contributions

JX and DZ conceived and designed the experiments. JX, LG, and WF performed the experiments. JJ, YG, and SC conducted the data analysis. HF and LG revised the manuscript. JX wrote the manuscript. All authors have read and approved the final version of this paper.

## Funding

This study was financially supported by the Natural Science Foundation of Liaoning Province (No.20170541028) and College Scientific Research Project of Education Department of Liaoning Province (No. LFWK201727).

## Conflict of Interest

The authors declare that the research was conducted in the absence of any commercial or financial relationships that could be construed as a potential conflict of interest.
